# Association Between Caregiver's Perception of “Good” Dietary Habits and Food Group Intake Among Preschool Children in Tokyo, Japan

**DOI:** 10.3389/fped.2019.00554

**Published:** 2020-01-22

**Authors:** Mayuko Kano, Yukako Tani, Manami Ochi, Noriko Sudo, Takeo Fujiwara

**Affiliations:** ^1^The Graduate School of Humanities and Sciences, Ochanomizu University, Tokyo, Japan; ^2^Department of Global Health Promotion, Tokyo Medical and Dental University, Tokyo, Japan; ^3^Department of Health and Welfare Services, National Institute of Public Health, Saitama, Japan; ^4^Natural Science Division, Faculty of Core Research, Ochanomizu University, Tokyo, Japan

**Keywords:** paternal perception, eating behavior, nutritional education, child nutrition, nutritional perception

## Abstract

Given that parents are mainly responsible for a preschooler's dietary management, they need to understand a child's diet. However, few studies have examined the association between parental perception of a preschool child's “good” dietary habits and actual food intake. We conducted a cross-sectional study investigating whether a child's food intake would differ depending on the caregiver's perception of their child's dietary habits among 4-year-old nursery school children at Adachi City, Tokyo, Japan. Children's dietary data were collected using the brief-type self-administered diet history questionnaire for children Aged 3–6 Years (BDHQ3y), while caregivers' perceptions of their child's dietary habits (good, normal, and poor) were inquired (*N* = 136). The percentage of caregivers who perceived their child's dietary habit as good, normal, and poor was 41.2, 40.4, and 18.4%, respectively. Multiple linear regression analysis revealed that children whose caregivers perceived their diet as poor showed lower intakes of vegetables [β = −48.7, 95% confidence interval (CI): −86.1 to −11.2], beans (β = −13.2, 95% CI: −26.1 to −0.3), and fish and shellfish (β = −9.2, 95% CI: −17.5 to −1.0) and higher intakes of fat and oil (β = 1.7, 95% CI: 0.4 to 3.1), confectionaries (β = 11.9, 95% CI: 3.6 to 20.3), and soft drinks (β = 31.2, 95% CI: 3.5 to 59.0) compared to children whose caregivers perceived their diet as good (all measures are in g/1,000 kcal per day). No significant difference was observed in other food groups, such as dairy products, an important source of protein and calcium for children. The current study may therefore guide future nutritional education programs for parents of preschool children.

## Introduction

Appropriate dietary habits are important for health not only during childhood but also throughout life considering that dietary habits during childhood are potentially inherited into adulthood (1). Therefore, constructing healthy dietary habits during childhood may lead to ideal dietary habits during adulthood, which could subsequently prevent diet-related diseases, such as cardiovascular diseases and diabetes, that account for 70% of the total deaths in 2016 worldwide (2).

One of the most important factors for children's dietary habits is their parents (3–7). Considering that preschool children are incapable of managing their own dietary habits (8), parental involvement and nutritional knowledge have been considered vital in establishing a child's ideal eating habits (9, 10). During preparation of preschool children's food, parental perception of the child's dietary habits, that is, their perception of “good” and “bad” foods for preschool children, is one of the key factors determining the child's diet. However, to the best of our knowledge, few studies have examined the association between caregivers' perception of good food for preschool children and the actual food intake of such children.

Some studies investigating adults' perception of their own diet have been published. Some of them have shown that adults hardly perceived their diet quality accurately with regard to both food group or nutrient intake and overall diet quality (11–14). In particular, adults whose dietary intake did not reach the recommended amount or those who did not intend to improve their dietary behavior were more likely to misperceive their diet quality (11, 12). Furthermore, although adults who perceive their diet as good tend to have an ideal nutrient intake compared to those who perceive their diet as normal or poor, they still did not meet the dietary intake recommendations (13). However, only a few studies have investigated the association between caregivers' perception of a child's dietary habits and actual food intake (15).

Although several studies have investigated general recognition of foods that are healthy or unhealthy among adults (16–18), no research has focused on what foods caregivers recognize as healthy or unhealthy for their child. A previous study investigating the association between a child's healthy eating index, assessed using a 24-h dietary recall, and maternal perception of the child's diet among Greek children aged 2–5 years old found that 83% of the mothers overestimated their child's nutritional status (15). In other words, most of the mothers perceived their child's diet as good when in fact the actual status of the child's diet was poor. Considering that young children are not able to manage their own diet and their caregivers have a large influence over the same, investigating the gap between caregivers' perception of dietary habits and actual food intake among young children can be of value. Nonetheless, no study has yet investigated the association between parental perceptions of a child's dietary habits and the child's quantitative food intake in Japan.

The present study therefore aimed to determine the association between caregivers' perception of a child's food intake and the actual child's food intake in Japan. In addition, we examined what food groups were more or less consumed when caregivers perceived their child's diet as good.

## Methods

### Study Design and Participants

This study used data on dietary habits among preschool children in Adachi City, Tokyo, Japan. The survey aimed to investigate and improve child's health as part of a diabetes prevention program implemented in Adachi City. All caregivers of 4-year-old children enrolled at seven licensed public nursery schools in Adachi City were invited to participate in the survey. The research was introduced on occasions when caregivers gathered at the nursery schools, such as during parent–teacher meetings, and questionnaires were distributed from January to February 2016. This study utilized two types of questionnaires: the brief-type self-administered diet history questionnaire (BDHQ) and a dietary habits questionnaire. Among the 154 child–caregiver pairs, 137 (response rate: 89%) submitted the answer sheets in sealed envelopes via each nursery school. Each respondent received a gift certificate equivalent to JPY 1000 (~USD 10). Among the 137 pairs, one child–caregiver pair was excluded from analysis for missing the main outcome, that is, caregivers' perception of child's dietary habits. Ultimately, 136 child–caregiver pairs were analyzed herein.

### Brief-Type Self-Administered Diet History Questionnaire

To assess the child's food intake, the BDHQ for preschool children aged 3–6 years (BDHQ3y) was used (19). While the BDHQ was developed to assess dietary intake during the preceding month among Japanese individuals (20), the BDHQ3y is a questionnaire that collects a child's anthropometric and habitual dietary data using the following four sections of inquiries: (i) intake frequency of 57 food and non-alcoholic beverage items; (ii) daily intake of rice (the most widely consumed staple food in Japan), including type of rice (refined, unrefined, etc.), and miso soup (traditionally consumed salty soup and widely considered to influence Japanese salt intake); (iii) usual cooking methods; and (iv) general dietary behavior (19, 20). The BDHQ3y has almost the same structure as the BDHQ for adults, although it has been modified to consider foods frequently consumed by children (19). For instance, the items, such as alcoholic beverages and coffee, are excluded, while other items frequently consumed by children, such as yogurt drinks, French fries, chocolate, and ketchup, are included. The BDHQ3y uses an age-specific fixed portion size for calculations, though information on portion sizes and calculation methods are not readily available (21). A more detailed description of the BDHQ and BDHQ3y, as well as their validity, has been published previously (19, 22). During BDHQ3y administration, children's caregivers fill out the answer sheets instead of the children. In case of missed questions, the BDHQ software originally substitutes answers following alternative values: the least frequency for questions regarding food consumption frequency and the median value for other questions, such as those relate to cooking method (23). The following food groups were considered in our analysis: grains, potatoes, vegetables, fruits, beans, fish and shellfish, meat, eggs, dairy products, oil and fat, confectionaries, soft drinks, teas, and 100% fruit and vegetable juices. The food classification used herein was based on a previous study (19) and is described in Table 1. The intake of each food group (g per day) was calculated by summing the intake of each food item as indicated in the BDHQ3y and dividing it by the total energy intake to obtain an energy-adjusted value (g/1,000 kcal per day), adopting the density method (24). Therefore, the individual food intake was presented as a dietary composition (i.e., a ratio of the daily energy intake).

**Table 1:** Classification of the food items indicated in the brief-type self-administered diet history questionnaire for children Aged 3–6 Years into food groups.

**Food group**	**Food item**
Cereals	Rice; Rice with sprinkle; Germinated/unrefined/multigrain/wheat-blended rice; Buckwheat noodles; Japanese wheat noodles; instant noodles and Chinese noodles; spaghetti and macaroni; Breads (including white bread and Japanese bread with a sweet filling)
Potatoes	Potatoes (including potatoes, sweet potatoes, taro, yam, other potatoes); french fries and potato chips
Vegetables	Carrots and pumpkins; tomatoes (including boiled tomato and stewed tomato); green leafy vegetables including broccoli; salted green and yellow vegetable pickles; raw vegetables used in salad (cabbage and lettuce); cabbage and Chinese cabbage; radishes and turnips; other root vegetables (onions, burdock, and lotus root); other salted vegetable pickles; mushrooms (all varieties); seaweeds (all varieties)
Fruits	Citrus fruit including oranges, strawberries; persimmons and kiwi fruit; other fruits; jam
Beans	Tofu (i.e., soybean curd) and tofu products; natto (i.e., fermented soybeans); miso for miso soup
Fish and shellfish	Dried fish and salted fish (including salted mackerel, salted salmon, and dried horse mackerel); small fish with bones; canned tuna; oily fish (including sardines, mackerel, saury, amberjack, herring, eel, and fatty tuna); non-oily fish (including salmon, trout, white meat fish, freshwater fish, and bonito); squid, octopus, shrimp and clam; fish meat paste products
Meat	Chicken (including ground chicken); pork and beef (including ground pork and beef); liver; ham, sausages, and bacon
Eggs	Eggs
Dairy products	Full-fat milk; low-fat milk; yogurt and yogurt drink; cheese; ice cream
Fat and oil	Butter; margarine; mayonnaise
Confectionaries	Rice crackers, rice cakes and Japanese-style pancakes; Japanese sweets; cakes, cookies and biscuits; snacks; chocolates
Soft drinks	Cola and sweetened soft drinks (including sports drinks); cocoa; lactic acid bacteria beverages; fruit juice excluding 100% juice
Tea	All kind of teas (including black and oolong tea)
Fruit and vegetable juice	100% fruit and vegetable juice

### Caregivers' Perception of Their Child's Dietary Habits

To assess caregivers' perception of their child's dietary habits, they were asked to answer the question “How would you characterize your child's diet?” by selecting one of the following five responses: good, fairly good, normal, not so good, and not good. Based on categories used in previous studies (15), these responses were classified into the following three groups: children whose caregivers perceived their dietary habits as “good” or “fairly good” comprised the good diet group, those perceived as “normal” comprised the normal diet group, and those perceived as “not so good” or “not good” comprised the poor diet group.

### Covariates

Data regarding the child's sex, presence of food allergies, height, and weight were collected from the BDHQ3y. The child's BMI-for-age z-score was calculated by inserting their height, weight, and age in months into the World Health Organization (WHO) Anthro or AnthroPlus software (25, 26). Data regarding caregivers' age, height, weight, and employment (self-employed, full-time job, part-time job, student, or others) were obtained via questionnaire. Caregivers' BMI, which was calculated using their self-reported height and weight data, was used to classify them into the following three groups according to the WHO cutoff: underweight (<18.5), normal (18.5–24.9), and overweight or obesity (≥25). Caregivers who had missing anthropometric data were classified into the missing group (*n* = 10).

### Statistical Analysis

Descriptive statistics was used to identify demographic data, while the chi-square test and analysis of variance was used to determine differences in demographic data according to caregivers' perception of child's dietary habits. Multiple linear regression analysis was conducted to determine whether food intakes differed according to caregivers' perception of their child's dietary habits. All categorical variables were converted into dummy variables and subsequently entered into the linear regression model. Firstly, caregivers' perception of their child's dietary habits was entered as a predictor of a child's food intake (Model 1). Thereafter, other covariates, including the child's sex, presence of food allergies, BMI-for-age z-score, and caregivers' age, employment, and BMI status were entered as simultaneous predictors (Model 2). To account for skewed intake of food groups, multiple linear regression was also conducted using logarithmic transformed values. All analyses were conducted using SPSS ver. 25 with the significance level set at 0.05.

### Ethics

The present study was conducted according to the latest guidelines provided in the Declaration of Helsinki and Ministry of Health, Labor and Welfare of Japan and had been approved by the Ethics Committee of the Tokyo Medical and Dental University (Approval No. M2016-284-02). Prior to data collection, information regarding the research had been provided to the participants through the nursery school principals. Those who opted not to participate in the research could decline to respond. Participants' submission of the research paper was considered as having provided consent to participate.

## Results

According to Table 2, almost all respondents were mothers (*n* = 133, 97.8%), two were fathers, and one was a grandmother. The children had an average age of 63.9 ± 3.6 months. Among the 136 children, 41.2, 40.4, and 18.4% were perceived as good, normal, and poor by their caregivers (respondents), respectively. Almost half of the children were male, while 10.3% had food allergies. The children had an average height, weight, and BMI of 108.1 cm, 18.2 kg, and 15.5 kg/m2, respectively, and had an average energy intake of 1368.9 kcal, which was close to the estimated energy requirement for 3–5 year-old Japanese children (27). Caregivers' average age was 35.7 (SD = 6.2) years. Approximately 45% of the caregivers were full-time workers, 40% were part-time workers, and 15% were self-employed workers and others. Although the good diet group seemed to comprise more full-time and fewer part-time workers compared to the other two groups, the differences were not significant. Almost all variables showed no significant differences according to caregivers' perception of their child's dietary habits, except for the caregiver's BMI status. The poor diet group, that is, caregivers who perceived their child to have a poor diet, had a higher prevalence of being overweight (44.0%) compared to the good and normal diet groups (7.1 and 16.4%, respectively).

**Table 2:** Characteristics of the 136 caregiver–child pairs according to caregivers' perception of their child's eating habits.

	**Caregiver's perception of child's dietary habits**								***p*-value^a^**
	**Total (*****N*** **= 136)**		**Good (*****n*** **= 56)**		**Normal (*****n*** **= 55)**		**Poor (*****n*** **= 25)**		
	**N (%)**		**%**		**%**		**%**		
**Child's sex**									
Male	64 (47.1)		46.4		45.5		52.0		0.856
Female	72 (52.9)		53.6		54.5		48.0		
**Whether child has food allergies**									0.596
Yes	14 (10.3)		7.1		12.7		12.0		
No	122 (89.7)		92.9		87.3		88.0		
**Caregiver's employment**									0.442
Self employed	9 (6.6)		8.9		5.5		4.0		
Full-time job	61 (44.9)		53.6		38.2		40.0		
Part-time job	57 (41.9)		30.4		49.1		52.0		
Others	9 (6.6)		7.1		7.3		4.0		
**Caregiver's BMI**									**0.010**
Underweight (<18.5)	14 (10.3)		10.7		10.9		8.0		
Normal (18.5–24.9)	88 (64.7)		75.0		65.5		40.0		
Overweight or obesity (25 ≤)	24 (17.6)		7.1		16.4		44.0		
Missing	10 (7.4)		7.1		7.3		8.0		
	**Mean**	**SD**	**Mean**	**SD**	**Mean**	**SD**	**Mean**	**SD**	
Child's BMI-for-age *z*-score	0.12	0.93	0.12	0.90	0.01	1.01	0.35	0.82	0.304
Child's energy intake (kcal)	1368.9	441.6	1370.8	364.5	1378.7	490.0	1338.2	501.2	0.930
Caregiver's age (year)	35.68	6.18	36.95	5.78	34.78	6.64	34.84	5.74	0.137

a *Chi-square test for frequency data and ANOVA for continuous variables. Bold values indicate the presence of significant difference (p < 0.05)*.

Descriptive data of the children's food intake and regression coefficients according to caregivers' perception of their child's dietary habits are presented in Table 3. Children perceived to have a better diet by their caregivers consumed larger amounts of certain food groups, such as vegetables, beans, fish and shellfish, and eggs, and lesser amounts of oil and fats, confectionaries, and soft drinks, compared to those perceived to have a poorer diet. Multiple linear regression analysis adjusting for simultaneous predictors (Model 2) showed significant differences between the good and poor diet groups in terms of the intake amount of seven out of 14 food groups: vegetables, fruits, beans, fish and shellfish, oil and fats, confectionaries, and soft drinks. Accordingly, children in the good diet group consumed higher amounts of vegetables, fruits, beans, and fish and shellfish (regression coefficient of the poor diet group relative to the good diet group: −48.7, −8.1, −0.23, and −0.24 g, respectively) but lower amounts of oil and fat, confectionaries, and soft drinks compared to those in poor diet group (regression coefficient of the poor diet group relative to the good diet group: 1.7, 11.9, and 31.7 g, respectively). Moreover, the normal diet group had a significantly lesser bean consumption and more confectionary intake compared to the good diet group. Considering that the intake of some food groups was skewed, multiple linear regression analysis was repeated using logarithmic transformed values. Nonetheless, similar results were obtained. Supplementary Table 1 shows the children's intake of five nutrients that are important for child health. The values of intakes were adjusted for each child's estimated energy requirement. Although all children reached the recommended dietary allowance (RDA) of protein, for other nutrients such as dietary fiber and calcium, most of children did not reach the RDA, even children in good diet group. Among good diet group, the percentage of children who did not meet the RDA was 67.9% for dietary fiber, 62.5% for calcium, 5.4% for n-3 fatty acid, 42.9% for iron.

**Table 3:** Multiple linear regression analysis of children's food intake (g/1,000 kcal per day) according to caregivers' perception of their child's diet.

**Food group**	**Caregiver's perception**	**Child's energy–adjusted food intake**^a^		**Model 1 β (95%CI)^a^**	**Model 2 β (95%CI)^a^**
		**Mean**	**SD**		
Grains	Good	238.1	56.2	Reference	
	Normal	242.3	70.6	4.2 (−20.8 to 29.2)	2.7 (−24.0 to 29.4)
	Poor	215.7	78.1	−22.4 (−54.1 to 9.3)	−15.8 (−51.8 to 20.2)
Potatoes	Good	19.5	16.1	Reference	
	Normal	15.8	10.1	−3.7 (−8.5 to 1.2)	−3.6 (−8.5 to 1.3)
	Poor	14.5	9.6	−4.9 (−11.1 to 1.2)	−5.3 (−11.9 to 1.4)
Vegetables	Good	119.6	68.1	Reference	
	Normal	86.4	82.8	−23.2 (−49.5 to 3.1)	−19.7 (−47.5 to 8.0)
	Poor	69.9	33.3	−49.7 (−83.0 to −16.4)^*^	−48.7 (−86.1 to −11.2)^*^
Fruits	Good	24.9	14.0	Reference	
	Normal	22.2	15.2	−2.7 (−7.9 to 2.5)	−2.8 (−8.1 to 2.5)
	Poor	16.8	9.5	−8.1 (−14.7 to −1.5)^*^	−8.1 (−15.2 to −1.0) ^*^
Beans	Good	61.1	35.6	Reference	
	Normal	52.3	29.5	−10.6 (−19.6 to −1.5)^*^	−10.5 (−20.0 to −0.9)^*^
	Poor	62.6	54.5	−11.2 (−22.6 to 0.3)	−13.2 (−26.1 to −0.3)^*^
Fish and shellfish	Good	30.9	15.1	Reference	
	Normal	27.14	17.0	−3.8 (−9.6 to 2.1)	−4.4 (−10.5 to 1.7)
	Poor	22.6	13.0	−8.3 (−15.7 to −0.9)^*^	−9.2 (−17.5 to −1.0)^*^
Meat	Good	32.2	12.2	Reference	
	Normal	31.7	13.3	−0.6 (−5.3 to 4.2)	−1.0 (−5.9 to 4.0)
	Poor	32.1	12.1	−0.2 (−6.2 to 5.8)	0.3 (−6.3 to 6.9)
Eggs	Good	13.5	9.9	Reference	
	Normal	12.2	9.6	−1.2 (−4.9 to 2.4)	−1.3 (−5.1 to 2.5)
	Poor	10.7	9.2	−2.8 (−7.3 to 1.8)	−2.3 (−7.4 to 2.8)
Dairy products	Good	146.2	84.9	Reference	
	Normal	123.1	79.3	−23.1 (−55.8 to 9.5)	−23.1 (−56.5 to 10.2)
	Poor	141.4	106.5	−4.8 (−46.3 to 36.6)	−15.0 (−60.0 to 30.0)
Fat and oil	Good	5.4	2.2	Reference	
	Normal	6.1	2.8	0.6 (−0.4 to 1.6)	0.5 (−0.5 to 1.5)
	Poor	7.5	2.8	2.0 (0.8 to 3.3)^**^	1.7 (0.4 to 3.1)^*^
Confectioneries	Good	19.2	14.1	Reference	
	Normal	27.9	16.6	8.7 (2.7 to 14.7)^**^	9.4 (3.2 to 15.7)^**^
	Poor	31.0	18.1	11.8 (4.2 to 19.4)^**^	11.9 (3.6 to 20.3)^**^
Soft drinks	Good	18.6	25.5	Reference	
	Normal	38.9	36.1	20.3 (0.9 to 39.8)^*^	17.5 (−3.1 to 38.1)
	Poor	55.2	66.6	36.6 (11.9 to 61.2)^**^	31.2 (3.5 to 59.0)^*^
Tea	Good	98.1	104.1	Reference	
	Normal	97.8	100.2	−0.4 (−37.6 to 36.8)	−2.7 (−41.6 to 36.3)
	Poor	105.5	83.4	7.4 (−39.7 to 54.5)	2.8 (−49.7 to 55.3)
100% juice	Good	16.9	22.5	Reference	
	Normal	19.3	26.7	2.4 (−12.1 to 16.9)	0.2 (−13.8 to 14.2)
	Poor	33.3	74.3	16.4 (−2.0 to 34.7)	9.0 (−9.9 to 27.8)

*Model 1: Univariate Liner regression that assessed caregiver's perception*.

Model 2: Adjusted for child's sex, BMI, food allergies, caregiver's age, employment and BMI.

a *Values are described as g/1,000 kcal per day*.

* p < 0.05,

** *p < 0.01. Bold values indicate the presence of significant difference (p < 0.05)*.

## Discussion

The present study investigated the association between caregivers' perception of their child's “good” dietary habits and the child's actual food intake. Multiple linear regression revealed that children whose diet qualities were perceived as “good” by their caregivers had significantly higher intakes of vegetables, beans, and fish and shellfish than those whose diet qualities were perceived to be poor, indicating that caregivers perceived their child's diet as “good” when the child consumed more of the aforementioned food groups. Such a finding can be considered reasonable given previous studies suggesting that individuals tend to recognize food high in fiber and low in energy content, such as vegetables, fruits, and beans, to be healthy (16–18). Similarly, results regarding fish and shellfish are not surprising given that the Japanese population generally recognizes these foods as healthy owing to their low fat content, being an excellent source of protein, and good omega-3 fatty acid content, which reduces cholesterol levels, contribute to the prevention of cardiovascular diseases, and enhance cognitive development (28–31).

Our results also showed the children perceived to have a good diet had lower intakes of oil and fats, confectionaries, and soft drinks, indicating that caregivers perceived their child's diet as good when their child consumed less of the aforementioned food group. Accordingly, the results presented herein are consistent with those reported in previous studies wherein foods high in fat and sugar contents were recognized as unhealthy (16–18).

No difference in dairy product consumption had been observed according to the caregivers' perception of their child's dietary habits. This is surprising considering that such food groups are supposed to have a large influence on childhood diet and health given children's increased need for calcium from dairy products to support bone growth as they grow older (32) and their higher calcium requirement relative to adults (27, 33). Our study realized that estimated calcium intake did not reach the recommend dietary allowance in all groups, including the good diet group (Supplementary Table 1). Thus, it is recommended that children consume appropriate amounts of dairy products.

No difference in the intake of meat and eggs, typically characterized as rich protein sources, had been observed according to caregivers' perception, indicating that caregivers pay relatively little attention to protein intake. Although fish and beans, which appear to be considered by caregivers when perceiving their child's diet quality, are also excellent protein sources, interest in such foods may be unrelated to their protein content but instead to other nutritional characteristics, such as their low energy, high fiber, and specific nutrient content. Additionally, previous research has also reported that when individuals evaluate their diet quality, they tend to neglect protein content unlike fiber and energy content (17). This is problematic considering that children require substantial amounts of protein for their rapid growth, similar to calcium. Although children included in present study had adequate protein intake (Supplementary Table 1), caregivers must change their perception toward protein, placing increased importance therein. Moreover, meat has been considered an important source of iron, which is also required for preventing anemia among children (34). Although some vegetables do contain iron, meat provides heme iron that is highly bioavailable (35).

Not only the difference of food intake among participants, but also the compliance with recommendations, is important. Many children did not achieve the RDA for some nutrients (Supplementary Table 1). It indicates that even if the child is perceived to have good dietary habits by their caregivers, their intake of some nutrients may not be enough. This fact reminds us the importance of taking care of both quality and quantity of food intake.

In summary, our results showed that although caregivers were able to recognize the importance of supposedly healthy foods, such as vegetables, they did not consider other foods, such as dairy products. Moreover, many children did not reach the RDA for some nutrients regardless of dietary habit. Hence, the importance and the appropriate intake amount of each food group should be emphasized among caregivers.

This study has several limitations worth noting. First, considering our focus on only public nursery school children, most mothers included herein were working, precluding generalizability to children of housewives. Also, we could not identify which caregiver was mainly in charge of the food selection for their children. However, national survey in Japan showed that 85.5% of housework including meal preparation and 80.6% of childcare including child feeding were owed by mothers (36). Therefore, in this study setting, it is estimated that most respondents are also in charge of preparing meals. Second, response bias might have induced misclassification errors in caregivers' perception, particularly with the use of a single-item questionnaire. However, this had been the primary method of measuring diet quality perception in previous studies (12). Third, the BDHQ3y carries low validity for potato and meat intake (19). Therefore, the relationship between caregivers' perception and their child's diet with regard to potatoes and meat may not have been appropriately assessed. Fourth, participants' socioeconomic status, which is strongly related to food intake (37–39), was not determined. Instead, we utilized caregivers' occupation in place of socioeconomic status during analysis. Fifth, given that anthropometry data were self-reported by the respondents, the accuracy of such data may be questionable. Sixth, we did not identify the extent of parents' wrong estimation of their children's diet quality and the way that this could influence the result analysis. Finally, the present study was cross-sectional in nature and could not determine a causal association between caregivers' perception and child's dietary habits, that is, the child's baseline food preference was not considered. Considering that a causal association may provide more information necessary for understanding caregivers' perception of their child's diet and providing better caregiver education, this should be considered in future studies.

In conclusion, caregivers perceived their child's diet as “good” when their child consumed high amounts of supposedly healthy foods, such as vegetables, fruits, beans and fish, and low amounts of supposedly unhealthy foods, such as oil and fat, confectionaries, and soft drinks. However, other food groups, such as dairy products, had not been considered despite being important nutrient sources for children's ideal growth. Therefore, caregivers should understand and pay more attention to the importance of these other food groups. Individuals responsible for caregiver education, such as local dietitians and nutrition program directors, should emphasize the importance of other food groups in addition to the already known healthy food groups.

## Data Availability Statement

The datasets generated for this study will not be made publicly available. The Ethics committee did not give permission for the data to be made publicly available.

## Ethics Statement

The studies involving human participants were reviewed and approved by Ethics Commitee of the Tokyo Medical and Dental University. Written informed consent from the participants' legal guardian/next of kin was not required to participate in this study in accordance with the national legislation and the institutional requirements.

## Author Contributions

MK, YT, and TF conceived the design. YT, MO, and TF collected data. MK and YT reviewed literature. MK analyzed data and wrote first draft of paper. YT revised the first draft. MO, NS, and TF edited the manuscript. All authors approved the final version of the manuscript.

### Conflict of Interest

The authors declare that the research was conducted in the absence of any commercial or financial relationships that could be construed as a potential conflict of interest.
