# Supplementary material for: Association Between Caregiver's Perception of “Good” Dietary Habits and Food Group Intake Among Preschool Children in Tokyo, Japan
Source: Front Pediatr. 2020 Jan 22;7:554. doi: 10.3389/fped.2019.00554 (PMC6987261; doi:10.3389/fped.2019.00554)
Supplement: Supplementary file 1 [file Data_Sheet_1.PDF]

Supplementary Table 1 | Comparison between recommended dietary allowance (RDA) [a] and observed children's energy-adjusted nutrient intake [b]

Nutrients	Recommended Dietary Allowance [a]	Total (N = 136)				Good (n = 56)				Normal (n = 55)				Poor (n = 25)			
		mean	SD	Median	Deficiency[c] (%)	mean	SD	Median	Deficiency[c] (%)	mean	SD	Median	Deficiency[c] (%)	mean	SD	Median	Deficiency[c] (%)
**Protein (g/EER day)**	25g	46.7	7.6	46.9	0.0	49.0	7.0	48.9	0.0	45.9	8.0	45.3	0.0	43.6	7.3	44.6	0.0
**Dietary fiber (g/EER per day)**	10g [d]	8.6	2.4	8.3	78.7	9.1	2.3	9.3	67.9	8.4	2.7	8.0	81.8	7.6	1.2	7.6	96.0
**Calcium (mg/EER per day)**	575mg [e]	494.0	152.3	471.6	74.3	535.4	130.2	543.8	62.5	469.4	166.0	439.1	83.6	459.0	155.5	454.7	80.0
**n-3 fatty acid (g/EER per day)**	1.2g [e]	1.6	0.4	1.5	12.5	1.6	0.4	1.6	5.4	1.5	0.5	1.4	21.8	1.6	0.3	1.6	8.0
**Iron (mg/EER per day)**	5.25mg [e]	5.2	1.2	5.1	55.1	5.5	1.1	5.6	42.9	5.2	1.3	5.1	60.0	4.7	0.7	4.8	72.0

[a] Referred to Japanese Dietary Reference Intakes 2015.(27)

[b] Children's nutrient intakes were adjusted for each child's estimated energy requirement (EER).

[c] Percentage of children who did not meet the RDA

[d] RDA of dietary fiber for 4-5 year-old children have been not clearly estimated, so we substituted DG (tentative dietary goals for preventing lifestyle-related diseases) for 6-7 year-old children.

[e] The mean value of RDA for male and female.
